# Gender-specific associations between fat mass, metabolic syndrome and musculoskeletal pain in community residents: A three-year longitudinal study

**DOI:** 10.1371/journal.pone.0200138

**Published:** 2018-07-09

**Authors:** In Young Park, Nam Han Cho, Seung Hun Lim, Hyun Ah Kim

**Affiliations:** 1 Department of Internal Medicine, Hallym University Sacred Heart Hospital, Anyang, Korea; 2 Department of Preventive Medicine, Ajou University School of Medicine, Suwon, Korea; 3 Institute for Skeletal Aging, Hallym University, Chunchon, Korea; University of Oslo, NORWAY

## Abstract

Increase in fat mass is correlated with musculoskeletal pain. The aim of this study was to examine the relationship between fat mass and the musculoskeletal pain prospectively in Korean community residents. In the Korean Health and Genome Study, participants (mean age 60.2 years, 56.2% women) completed pain questionnaires and underwent dual x-ray absorptiometry to calculate body composition. Three-year follow-up data on pain was available for 1,325 participants. Pain was categorized according to number of pain regions. At three years of follow-up, participants were classified as follows: 1) no pain both at baseline and at three years (no pain), 2) any pain (one, two or more, or widespread regions) at baseline and no pain at three years (transient pain), 3) no pain at baseline and any pain at three years (new pain) 4) any pain both at baseline and at 3 years (persistent pain). 1) and 2) were grouped as no/transient pain group (no pain) and 3) and 4) as new/persistent pain group (pain). Female gender and obesity were two significant factors associated with the persistence or development of pain. Total fat mass and fat:muscle mass ratio were associated with pain among female participants only, and the odds ratios for pain were significantly increased in female participants in the highest quartile of total fat mass and fat muscle ratio after adjustment. In conclusion, both female gender and obesity were two significant factors associated with pain. Fat mass parameters and pain were significantly associated only among females.

## Introduction

Low back, neck and other musculoskeletal pain, ranked the first, fourth and sixth in the leading causes of years lived with disability (YLD) globally [1]. It, thus, causes a major burden on individuals, health systems, and social care systems, and is expected to become the most important public health challenge with the population aging. The reported prevalence of musculoskeletal pain varies, however, the rate of chronic widespread pain, the most serious category, is rather consistently reported to be around 12% [2]. Musculoskeletal pain results from multiple, heterogeneous pathologies including osteoarthritis (OA), tendinitis/bursitis and fibromyalgia[3]. Risk factors of musculoskeletal pain, thus, are varied ranging from genetic factors to demographic factors including old age and female sex, and environmental factors including high physical workload, psychosocial distress, and low physical activity [4]. Obesity is a strong risk factor of OA, musculoskeletal pain and physical dysfunction. Specifically, studies assessing body mass index (BMI) consistently showed it to be a risk factor for knee OA, with the random-effects pooled odds ratio for overweight or obese compared to normal weight being 2.96 in one meta-analysis [5]. In addition, increasing levels of pain are observed across the continuum of BMI classifications, from low-normal BMI through BMI ≥40 [6]. Both obesity and musculoskeletal pain are serious health risk conferring increase in morbidity and mortality [7,8] thus it would be important to delineate the pathogenetic mechanism linking the two conditions.

Traditionally the relationship between obesity and pain has simply been regarded as resulting from the intermediary effect of arthritis due to increased joint loading [9]. Currently the adipose tissue is considered to be an endocrine organ promoting low-grade systemic inflammation by secreting adipokines [10]. In an 11-year Norwegian cohort study of general population, obese participants had a 66% increased odds of the persistence of chronic widespread pain compared with normal weight individuals [11]. Previous reports have used BMI, waist circumference and waist-hip index as measures of obesity, which do not represent specific components of body composition. Previously, we have shown that increase in fat mass and fat/muscle mass ratio was significantly associated with musculoskeletal pain among women [3]. Higher prevalence of widespread pain was observed among those with metabolic syndrome in both normal and high BMI participants, implicating an independent influence of inflammation aside from mechanical factors [3]. In a study of 133 participants, who ranged from normal weight to obese, greater fat mass and fat mass index were associated with a greater number of lower body pain sites, with no association observed for fat-free mass [12]. However, a cross-sectional design of these studies failed to prove the causal role of fat mass.

In this study, we examined the relationship between fat mass and the musculoskeletal pain in three year prospective follow-up of Korean community residents. A gender difference in the relationship between fat mass and pain was examined. Additionally, the relationship between metabolic syndrome and musculoskeletal pain was evaluated by examining the risk for musculoskeletal pain stratified by BMI and the presence or absence of metabolic syndrome.

## Materials and methods

### Study population

In the ongoing prospective Korean Health and Genome Study, a rural farming community (Anseong) in Korea was selected. Anseong, a county approximately 70 km south of Seoul, had a population of 132,906 in 2000 [13]. The methods of this study have been previously described elsewhere [3]. Briefly, the eligibility criteria included an age of 40 to 79 years, residence within the borders of the survey area for at least six months before testing, and the mental and physical ability to participate. Participants were invited both by the telephone and mail to the study with the announcement that “This is a study evaluating general health and physical function in the elderly.” Pain or arthritis was not mentioned in the study advertisement. From year 2008, dual x-ray absorptiometry (DEXA) examination began, and 1530 participants surveyed in year 2008–2009 went through DEXA examination with measurement of fat and muscle mass. After three years, 105 patients refused to participate in the follow-up survey, 22 patients developed other serious illness precluding participation in the survey, 42 participants died, 28 participants were lost, and 8 participants moved out of the survey area, leaving 1325 participants with available data in year 2011–2012. Although subjects who participated had significantly higher BMI compared to those who did not(24.36±3.23 vs 23.99±3.24), there were no significant differences in age or gender. Those who participated were more likely to be married, have higher level of education, and less likely to have DM or to smoke (S1 Table).

The ethics committees of the Korean Health and Genome Study and Ajou University School of Medicine approved the study protocol (approval number AJIRB-CRO-07-012). Written informed consent was obtained from each participant.

### Baseline data and health interview

Demographic information was collected at baseline that included educational attainment, occupation, exercise, and history of arthritis using a standard questionnaire during a face-to-face interview. Educational attainment was dichotomized into ≥ 12 years (finished high school, finished vocational school, some college, finished college, some graduate school and higher) or < 12 years for the analysis. The exercise category was self-reported and classified as none versus at least once per week (once/week, two-three times/week, and daily) for the analysis. Information on medication was collected, and those who were taking anti-hypertensives and anti-diabetic drug were defined as having hypertension and diabetes, respectively. History of hand or knee arthritis was self-reported by responding to the following question, “Have you ever been diagnosed as hand (or knee) arthritis by a physician?” Subjects also filled out the SF-12 questionnaire, which measures self-reported health status and quality of life.

### Anthropometric and laboratory measurement

Height (cm) and body weight (kg) were measured to the nearest 0.1 cm and 0.1 kg, respectively, with the subject wearing light clothing and barefooted for calculation of the BMI. Body mass index ≥ 27 kg/m^2^ was used as a criterion for obesity. DEXA (^®^Prodigy, GE Healthcare) was used to assess body composition. Total and regional (trunk, android, gynoid, and leg) fat masses were measured and analyzed by Encore Software 11(Encore Software Inc., Minneapolis, MN) in accordance with the manufacturer’s instruction. Briefly, trunk fat was designated from the pelvis cut (lower boundary) to the neck cut (upper boundary) and android fat was defined from the pelvis cut to above the pelvis cut by 20% of the distance between the pelvis and neck cuts. Gynoid fat was described from the lower boundary of the umbilicus to a line equal to twice the height of the android fat distribution. Plasma glucose concentrations were measured using the hexokinase method (ADVIA 1800 Auto Analyzer, Siemens Healthcare GmbH, Erlangen, Germany). The presence of diabetes mellitus (DM) was defined as either a fasting glucose level ≥ 126 mg/dL or a 2-hours glucose level of ≥ 200 mg/dL after 75 g oral glucose loading. Triglyceride and HDL cholesterol were measured by using ADVIA 1800 Auto Analyzer(Siemens Healthcare GmbH, Erlangen, Germany) based on the Fossati three-step enzymatic reaction and direct HDL cholesterol method with a Trinder reaction, respectively.

### Determination of metabolic syndrome components and classification of participants

Metabolic syndrome was identified by using the International Diabetes Federation 2005 recommendations (waist circumference > 90 cm in males, > 80 cm in females, serum triglyceride ≥ 150 mg/dL, high-density lipoprotein (HDL) cholesterol < 40 mg/dL in males, < 50 mg/dL in females, fasting blood glucose ≥ 100 mg/dL, and hypertension) [14]. The presence of hypertension was defined as either a systolic pressure ≥ 140 mmHg or a diastolic pressure ≥ 90 mmHg after measuring the blood pressure 3 times with a sphygmomanometer, with the second and third of three measurements averaged to estimate systolic and diastolic pressure. Those who were taking anti-hypertensives were defined as having hypertension. Participants were categorized into four groups as follows. Metabolically obese/normal weight(MO/NW) was defined as the presence of more than three features of metabolic syndrome and normal BMI (18.5 ≤ BMI < 25 and number of metabolic syndrome feature (MSf) ≥ 3). Three other groups were as follows: metabolically normal normal weight (MN/NW, 18.5 ≤ BMI < 25 and MSf ≤ 2), metabolically normal obesity (MN/OB, BMI ≥ 25 and MSf ≤ 2), and metabolically obese obesity (MO/OB, BMI ≥ 25 and MSf ≥ 3).

### Determination of pain categories

The measure of pain used in this study was based on location of pain, as Leveille et al reported previously[15]. In short, participants were asked if they had pain, aching, or stiffness in any of their joints on most days. Persons who responded ‘yes’ were asked to indicate painful area with circles on a homunculus depicting upper and lower extremity joints and four areas of the back and neck [16]. Pain was classified according to number of pain regions, and the most serious category was widespread pain, defined as pain above the waist, below the waist, on both sides of the body, and in the axial region according to the American College of Rheumatology criteria [17]. Three other categories of pain were pain in two or more regions that did not fulfill the definition for widespread pain, pain in one region, and no pain. After three years of follow-up, participants were further categorized as follows: 1) no pain both at baseline and at three years(no pain), 2) any pain (one, two or more, or widespread regions) at baseline and no pain at three years(transient pain), 3) no pain at baseline and any pain at three years(new pain) 4) any pain both at baseline and at three years(persistent pain). 1) and 2) were grouped as no/transient pain group(no pain group) and 3) and 4) as new/persistent pain group(pain group).

### Statistical analysis

Continuous variables were tested using Student’s t-test, and categorical variables were tested using Pearson’s chi-square test. Tests for correlation between pain and body composition were performed using ANCOVA after adjustment of age. The association between pain and demographic factors was analyzed using multivariable logistic regression analysis after adjusting for the factors significantly associated with pain in univariable analysis. The association between pain and fat/muscle mass ratio was analyzed using multivariable logistic regression analysis after adjusting for age, gender, and history of arthritis. The difference in the distribution of pain categories in MN/NW, MO/NW, MN/OB, and MO/OB was examined with Pearson’s chi-square test. Statistical analysis was performed with SPSS 12.0 (SPSS Inc., Chicago, IL, USA). P value < 0.05 (two-tailed) was considered statistically significant.

## Results

### Characteristics of the study participants

The mean age of participants (1,325, 580 men, 745 women) was 60.2 years (Table 1). Women had a higher BMI, higher rate of obesity and self-reported arthritis compared to men. Three hundred twenty two (24.3%) and 296(22.3%) of participants were grouped as no pain and transient pain group, respectively, and 268(20.2%) and 439(33.1%) as new and persistent pain group, respectively.

**Table 1:** Demographic characteristics.

	Men (N = 580)	Women (N = 745)	*p*
Age(yr)	59.96±8.22	60.44±8.56	0.302
BMI(kg/m^2^)	23.93±3.01	24.69±3.36	0.000
Obesity	86(14.8)	166(22.3)	0.001
Married	554(95.5)	598(80.3)	0.000
Education > 12years	242(41.7)	125(16.8)	0.000
Alcohol	397(68.4)	184(24.7)	0.000
Smoking	194(33.5)	11(1.5)	0.000
Exercise	253(43.6)	280(37.6)	0.026
Diabetes mellitus	119(20.6)	139(18.7)	0.390
Hypertension	105(18.1)	143(19.2)	0.613
Self-reported hand or knee arthritis	26(4.5)	112(15.0)	0.000
Manual work	112(19.3)	273(36.6)	0.000
MCS	71.61±12.96	64.84±15.56	0.000

Values are the mean ± SD for continuous variables and the number (%) for categorical variables. N = number of participants; BMI = body mass index; MCS = mental component summary of SF12. Obesity = BMI ≥ 27 kg/m^2^. Continuous variables were tested using Student’s t-test, and categorical variables were tested using Pearson’s chi-square test.

### Risk factors associated with new/persistent pain

We found out that female gender, and obesity were significantly associated with new or persistent pain after multivariable logistic regression analysis (data not shown). We then compared the body composition features and demographics between pain groups according to gender (Table 2).

**Table 2:** Demographic features of pain and no pain group stratified by gender.

	men			Women		
	no pain (N = 309)	New or persistent pain (N = 271)	*p*	no pain (N = 309)	New or persistent pain (N = 436)	*p*
Mean (SD)						
Age(yr)	59.62±8.02	60.35±8.43	0.289	59.08±8.25	61.40±8.66	<0.001
Body mass index (kg/m^2)^	23.81±3.00	24.06±3.03	0.318	24.22±3.15	25.02±3.46	0.001
Fat Mass(kg)	13.72±5.85	14.10±5.59	0.428	18.69±5.56	19.93±6.25	0.005
Lean Mass(kg)	50.53±5.88	50.48±6.02	0.923	36.55±3.63	36.20±3.86	0.211
Fat/muscle ratio	0.27±0.11	0.28±0.11	0.362	0.51±0.14	0.55±0.16	0.001
Percent						
Married	95.1	95.9	0.644	82.8	78.4	0.136
Education	44.0	39.1	0.233	19.4	14.9	0.105
Obesity	14.9	14.8	0.966	16.5	26.4	0.001
Alcohol	72.2	64.2	0.040	24.9	24.5	0.906
Smoking	34.7	32.1	0.502	1.6	1.4	0.787
Exercise	45.0	42.1	0.480	41.1	35.1	0.095
Diabetes mellitus	20.2	21.1	0.786	17.8	19.4	0.582
Hypertension	17.8	18.5	0.839	16.8	20.9	0.167
Manual work	20.4	18.1	0.482	40.8	33.7	0.049
Hand or knee arthritis	3.2	5.9	0.121	11.3	17.7	0.017

Continuous variables were tested using Student’s t-test, and categorical variables were tested using Pearson’s chi-square test.

While in women, new/persistent pain group had higher baseline BMI, fat mass and fat/muscle ratio, body composition features did not differ significantly in men according to pain status. New/persistent pain group was significantly older, less likely to do manual work, and had higher prevalence of self-reported hand or knee arthritis at baseline, only in women. Total fat mass and fat/muscle ratio were significantly and positively associated with new/persistent pain while total lean mass was significantly and negatively associated with it after adjustment of age (Table 3). However, total fat mass and fat/muscle ratio were significantly and positively associated with pain only among women.

**Table 3:** Correlation between fat mass and pain after adjustment for age.

	No pain Mean ± SE	New or persistent pain Mean ± SE	*p*
Male			
Total fat mass(kg)	13.69±0.32	14.13±0.35	0.345
Total lean mass(kg)	50.43±0.31	50.59±0.33	0.721
Fat/muscle ratio	0.27±0.01	0.28±0.01	0.340
Female			
Total fat mass(kg)	18.55±0.34	20.03±0.28	0.001
Total lean mass(kg)	36.38±0.21	36.32±0.17	0.815
Fat/muscle ratio	0.51±0.01	0.55±0.01	<0.001

Tests for correlation between pain and body composition were performed using ANCOVA after adjustment of age.

The association between each quartile of fat/muscle mass ratio and new/persistent pain was examined. Participants in the highest fat/muscle mass ratio quartile had significantly higher odds of pain, however the association was only significant among women. (Table 4). In addition, participants in the highest fat mass quartile had significantly higher odds of pain after adjustment, which was only significant among women (Table 5). Linear trend was significant for both fat/muscle ratio and fat mass after adjustment (data not shown).

**Table 4:** Association between each quartile of fat/muscle mass ratio and pain.

Fat/muscle mass ratio	Crude		Model 1		Model 2	
	OR (95% CI)	*p*	OR (95% CI)	*p*	OR (95% CI)	*p*
Quartile 1	-	-	-	-	-	-
Quartile 2						
Male	1.53(0.95–2.44)	0.08	1.55(0.96–2.48)	0.07	1.52(0.95–2.44)	0.08
Female	1.21(0.81–1.82)	0.35	1.23(0.81–1.75)	0.33	1.21(0.80–1.82)	0.37
Quartile 3						
Male	1.32(0.83–2.10)	0.25	1.34(0.84–2.14)	0.22	1.33(0.83–2.13)	0.23
female	1.26(0.84–1.89)	0.27	1.31(0.87–1.97)	0.2	1.28(0.84–1.93)	0.25
Quartile 4						
Male	1.33(0.83–2.13)	0.24	1.33(0.83–2.14)	0.23	1.31(0.82–2.11)	0.26
Female	2.02(1.32–3.09)	<0.01	2.05(1.34–3.15)	<0.01	1.98(1.29–3.05)	<0.01

Model 1 adjusted for age. Model 2 adjusted for age and arthritis.

**Table 5:** Association between each quartile of fat mass and pain.

Fat mass	Crude		Model 1		Model 2	
	OR (95% CI)	*p*	OR (95% CI)	*p*	OR (95% CI)	*p*
Quartile 1	-	-	-	-	-	-
Quartile 2						
Male	1.01(0.63–1.62)	0.97	1.03(0.64–1.66)	0.9	1.01(0.63–1.63)	0.97
Female	1.10(0.73–1.65)	0.66	1.18(0.78–1.79)	0.42	1.17(0.77–1.77)	0.47
Quartile 3						
Male	1.34(0.84–2.12)	0.22	1.37(0.86–2.19)	0.18	1.35(0.85–2.16)	0.21
female	1.24(0.82–1.87)	0.3	1.34(0.88–2.03)	0.17	1.30(0.85–1.98)	0.22
Quartile 4						
Male	1.11(0.70–1.77)	0.67	1.15(0.72–1.85)	0.56	1.13(0.70–1.81)	0.62
Female	1.54(1.02–2.35)	0.041	1.67(1.09–2.56)	0.02	1.62(1.06–2.48)	0.03

Model 1 adjusted for age. Model 2 adjusted for age and arthritis

### Association between metabolic syndrome and pain

The association between metabolic syndrome and pain was examined after classifying the participants into four groups, MN/NW, MO/NW, MN/OB, and MO/OB. The distribution of pain categories in each group was assessed. (Table 6). The prevalence of MN/NW, MO/NW, MN/OB, and MO/OB was 43.0, 16.1, 16.1 and 24.7% in this population. Among women, new/persistent pain was the least frequent in MN/NW group. However, the presence of metabolic syndrome did not increase pain among obese subjects. In addition, logistic regression analysis with BMI < 25 & MS ≤ 2 as the reference group showed that the risk of pain was not significantly increased in any of the group with or without BMI adjustment in both genders (Table 7).

**Table 6:** Distribution of pain after 3 years in 4 groups stratified by the presence and absence of metabolic syndrome and of obesity.

	Group	No pain	New or persistent pain (pain)	*p*
Male	BMI < 25 & MS ≤ 2	160(54.1)	136(45.9)	0.375
	BMI < 25 & MS ≥ 3	38(55.1)	31(44.9)	
	BMI ≥ 25 & MS ≤ 2	39(44.8)	48(55.2)	
	BMI ≥ 25 & MS ≥ 3	72(56.3)	56(43.8)	
	Total	309(53.3)	271(46.7)	
Female	BMI < 25 & MS ≤ 2	130(47.4)	144(52.6)	<0.05
	BMI < 25 & MS ≥ 3	61(42.4)	83(57.6)	
	BMI ≥ 25 & MS ≤ 2	45(35.7)	81(64.3)	
	BMI ≥ 25 & MS ≥ 3	73(36.3)	128(63.7)	
	Total	309(41.5)	436(58.5)	

Definition of MONW: normal BMI range (18.5 to 25 kg/m^2^) and presence of ≥ 3 metabolic syndrome features.

Values are the number (%). P values (for trend) were determined by Pearson’s chi-square test.

**Table 7:** Association between metabolic syndrome and pain in 4 groups stratified by BMI.

		OR (95% CI)	*P*	Adjusted OR (95% CI)	*P*
Male	BMI < 25 & MS ≤ 2	-	-	-	-
	BMI < 25 & MS ≥ 3	1.04(0.62–1.76)	0.878	1.11(0.65–1.90)	0.710
	BMI ≥ 25 & MS ≤ 2	1.51(0.80–2.85)	0.205	1.29(0.64–2.62)	0.478
	BMI ≥ 25 & MS ≥ 3	0.95(0.53–1.72)	0.874	0.80(0.41–1.59)	0.531
Female	BMI < 25 & MS ≤ 2	-	-	-	-
	BMI < 25 & MS ≥ 3	0.81(0.54–1.22)	0.322	0.88(0.58–1.34)	0.547
	BMI ≥ 25 & MS ≤ 2	1.32(0.81–2.16)	0.265	1.02(0.57–1.82)	0.954
	BMI ≥ 25 & MS ≥ 3	1.29(0.83–2.00)	0.257	0.96(0.55–1.68)	0.895

Adjusted OR was obtained after adjustment of BMI

## Discussion

In this three year prospective study examining the relationship between fat mass parameters and the musculoskeletal pain, female gender and obesity were two significant factors associated with the persistence or development of pain. After adjustment of age and the presence of arthritis, fat/ muscle ratio and fat mass were significantly associated with pain, which was only significant among females.

Musculoskeletal pain is a leading cause of disability globally affecting especially the elderly, and older women have a higher prevalence of pain as well as more disability from pain than older men [18]. Although OA, an important contributor to musculoskeletal pain in the elderly, is more common among women compared to men, it has been consistently reported that radiographic OA changes are poorly correlated with pain and physical function, thus additional factors explaining the gender difference in pain needs to be explored [19]. Previous study revealed that men and women differ in the factors associated with musculoskeletal pain, such that pain was associated with BMI, systolic blood pressure, and depressive symptoms in women while with polyarticular radiographic OA in men [15]. Body mass index is a risk factor for pain as well as OA, however, it is impossible to elucidate the cause and effect relationship between BMI and pain in cross sectional studies because pain may limit activity leading to increase in BMI. In the same line, although the fat mass parameters aside from BMI were reported as a significant factor associated with pain, lack of activity resulting from pain may influence fat mass more than BMI. Very recent reports began to reveal the relationship between adiposity and pain in a longitudinal setting. In participants aged 50–79 in Southern Tasmania, baseline BMI and body fat mass were deleteriously associated with consistent knee pain over 5.1 years follow-up [20]. Body mass index was associated with increases in weight-bearing and non-weight-bearing pain, while fat mass was associated with non-weight-bearing pain[20]. In the same cohort, participants reporting greater number of painful sites had greater fat mass, fat mass index, and BMI both cross-sectionally and longitudinally[21]. In a different cohort of participants ages ≥50 years from the North West Adelaide Health Study, the odds of having prevalent foot pain increased by 8% for each fat mass index unit, while the odds of having pain four years later increased by 6% for it [22]. Therefore, these three studies as well as ours strengthen the concept that body fat accounts for pain and is not merely the result of inanition due to pain. In pain studies, it is sometimes difficult to capture the pain status reliably in participants. For example, most people with symptomatic knee OA experience fluctuation in pain presence and severity [23]. Thus the definition of pain progression would be crucial to get to the conclusion. In our study, we included both participants who had no pain at baseline and any pain at three years(new pain) and who had pain both at baseline and at three years(persistent pain) as pain group. This definition might have introduced the shortcomings of cross-sectional analysis by including participants who already limited their activity due to pain at baseline. In a separate analysis comparing only those participants who did not have pain at both time points(n = 322) and those who developed pain at three years(new pain, n = 216), however, the result did not differ significantly with association of fat parameters and pain development at three years, which was only significant among females(S2–S4 Tables). Comparing no pain/resolved pain group vs persistent pain group led to the same result (significant association of fat mass and pain, which was only significant among females, S5 and S6 Tables). However, in the case comparing resolved pain group with the persistent pain group, most of the factors associated with pain disappeared (S7 and S8 Tables). We believe this is due to smaller sample size.

Our study shows stronger relationship between fat mass and pain among women, which suggest that fat mass parameter may be one mechanism explaining the increase in pain in women. Because women are reported to have a heightened inflammatory response compared with men, and fat tissue is considered an endocrine organ producing proinflammatory adipokines, stronger inflammatory response arising from increased fat in women may play a role in gender difference of pain [24]. In addition, estrogen deficiency after menopause which increases subcutaneous adipose tissue storage of free fatty acid may play a role linking increased fat mass and pain after menopause, since the literature is highly suggestive of the role of ovarian hormones in modulating pain [25,26].

The association between metabolic syndrome and pain was assessed by dividing the participants into four groups, MN/NW, MO/NW, MN/OB, MO/OB. Approximately 5–45% of lean individuals have metabolic abnormalities, which are linked to increased adipose tissue inflammation and low cardiorespiratory fitness [27]. Previously, we reported that compared to MN/NW participants, widespread pain was more common in MO/NW participants [3]. In this prospective study of the same population, we observed that female participants with MN/NW at baseline were less likely to belong to pain group three years later. However, logistic regression analysis with BMI < 25 & MS ≤ 2 as the reference group showed that the risk of pain was not significantly increased in any of the group with or without BMI adjustment in both genders. A previous report analyzing the association between the number of components of metabolic syndrome and incident symptomatic knee OA showed that the significant association was markedly attenuated and no longer significant after controlling for BMI [28]. Analysis using computed residuals of waist circumference by removing the variation caused by BMI or body weight still nullified the association, suggesting abdominal obesity, BMI, and body weight are measures of the same potential factor [28].

Our study is the first large-scale prospective study elucidating the relationship between fat mass and musculoskeletal pain among Asian subjects. Our study revealed the significant association of fat mass parameters and pain only among females, which may provide a clue to the gender difference in pain experience. Our study has limitations. The participants were recruited from a rural farming community and were aged 40–79 years. Thus, our result may not apply to urban population with different age range. We classified transient pain into the no pain group, which would have led to misclassification because it is possible that some of the new pain subjects would be classified as no pain group if they do not have pain after 3 more years of follow-up. We did not have detailed information about factors regulating both pain and fat mass, such as the amount of physical activity and psychological factors, failing to adjust for these confounders. Exercise was marginally and negatively associated with the prospective pain phenotype, however, this association disappeared after adjustment of confounders (data not shown). Lastly, our study only captured the presence of pain and not its intensity or character (nociceptive vs inflammatory, weight-bearing vs non-weight-bearing pain).

In conclusion, total fat mass and fat/ muscle ratio was significantly associated with pain and the association of fat mass parameters and pain was significant only among females.

## Supporting information

S1 Table Demographic characteristics of those who participated and those who did not. (DOCX)

S2 Table Correlation between fat mass and pain after adjustment for age (no pain group vs new pain group). (DOCX)

S3 Table Association between each quartile of fat/muscle mass ratio and development of pain (no pain group vs new pain group). (DOCX)

S4 Table Distribution of new pain after 3 years in 4 groups classified by the presence and absence of metabolic syndrome and of obesity. (DOCX)

S5 Table Correlation between fat mass and pain after adjustment for age (no pain/resolved pain group vs persistent pain group). (DOCX)

S6 Table Association between each quartile of fat/muscle mass ratio and pain(no pain/resolved pain group vs persistent pain group). (DOCX)

S7 Table Correlation between fat mass and pain after adjustment for age (resolved pain group vs persistent pain group). (DOCX)

S8 Table Association between each quartile of fat/muscle mass ratio and pain (resolved pain group vs persistent pain group). (DOCX)
