# Supplementary material for: Gender-specific associations between fat mass, metabolic syndrome and musculoskeletal pain in community residents: A three-year longitudinal study
Source: PLoS One. 2018 Jul 9;13(7):e0200138. doi: 10.1371/journal.pone.0200138 (PMC6037368; doi:10.1371/journal.pone.0200138)
Supplement: S1 Table — (DOCX) [file pone.0200138.s001.docx]

Supplementary Table 1. Demographic characteristics of those who participated and those who did not

|  | Those who participated  (N=1,325) | Those who did not participate  (N=205) | Total  (N=1,530) | p-value |
| --- | --- | --- | --- | --- |
| Obesity | 252(19.0) | 35(17.1) | 287(18.8) | 0.507 |
| Married | 1,152(86.9) | 166(81.0) | 1,318(86.1) | 0.021 |
| Education≥12 | 367(27.7) | 42(20.5) | 409(26.7) | 0.030 |
| Alcohol | 581(43.8) | 76(37.1) | 657(42.9) | 0.068 |
| Smoking | 205(15.5) | 46(22.4) | 251(16.4) | 0.012 |
| Exercise | 533(40.2) | 70(34.1) | 603(39.4) | 0.097 |
| Diabetes mellitus | 258(19.6) | 64(31.8) | 322(21.2) | <0.001 |
| Hypertension | 248(18.7) | 48(23.4) | 296(19.3) | 0.113 |
| Self-reported hand or knee arthritis | 138(10.4) | 18(8.8) | 156(10.2) | 0.472 |
| Manual work | 385(29.1) | 68(33.3) | 453(29.6) | 0.213 |
