# Supplementary material for: Gender-specific associations between fat mass, metabolic syndrome and musculoskeletal pain in community residents: A three-year longitudinal study
Source: PLoS One. 2018 Jul 9;13(7):e0200138. doi: 10.1371/journal.pone.0200138 (PMC6037368; doi:10.1371/journal.pone.0200138)
Supplement: S2 Table — (DOCX) [file pone.0200138.s002.docx]

Supplementary Table 2. Correlation between fat mass and pain after adjustment for age (No pain group vs new pain group)

|  | No pain (N=322) | New pain(N=216) | p-value |
| --- | --- | --- | --- |
|  | Mean ± SE | Mean ± SE |  |
| All |  |  |  |
| Total fat mass, kg | 15.1899±0.3281 | 17.0717±0.4007 | <0.001 |
| Total lean mass, kg | 44.7800±0.4882 | 43.3901±0.5963 | 0.072 |
| Fat/muscle ratio | 0.3531±0.0092 | 0.4148±0.0112 | <0.001 |
| Male |  |  |  |
| Total fat mass, kg | 13.8444±0.4030 | 14.2157±0.5559 | 0.589 |
| Total lean mass, kg | 50.3431±0.3788 | 51.5270±0.5226 | 0.068 |
| Fat/muscle ratio | 0.2732±0.0075 | 0.2724±0.0104 | 0.947 |
| Female |  |  |  |
| Total fat mass, kg | 17.2949±0.4799 | 19.6614±0.5070 | 0.001 |
| Total lean mass, kg | 36.0851±0.3238 | 36.0191±0.3421 | 0.889 |
| Fat/muscle ratio | 0.4782±0.0121 | 0.5435±0.0128 | <0.001 |

Tests for correlation between pain and body composition were performed using ANOCOVA after adjustment of age.
