# Supplementary material for: Gender-specific associations between fat mass, metabolic syndrome and musculoskeletal pain in community residents: A three-year longitudinal study
Source: PLoS One. 2018 Jul 9;13(7):e0200138. doi: 10.1371/journal.pone.0200138 (PMC6037368; doi:10.1371/journal.pone.0200138)
Supplement: S3 Table — (DOCX) [file pone.0200138.s003.docx]

Supplementary Table 3. Association between each quartile of fat/muscle mass ratio and development of pain (No pain group vs new pain group)

|  | Crude | |  | Model 1 | |  | Model 2 | |
| --- | --- | --- | --- | --- | --- | --- | --- | --- |
| Fat/muscle mass ratio | OR (95% CI) | *P* |  | OR (95% CI) | *P* |  | OR (95% CI) | *P* |
| Quartile 1 | - | - |  | - | - |  | - | - |
| Quartile 2 | 1.11(0.71-1.75) | 0.648 |  | 1.12(0.70-1.77) | 0.645 |  | 1.13(0.71-1.80) | 0.611 |
| Quartile 3 | 1.14(0.69-1.87) | 0.618 |  | 1.01(0.54-1.87) | 0.985 |  | 1.06(0.57-1.97) | 0.862 |
| Quartile 4 | 2.87(1.70-4.82) | <0.001 |  | 2.47(1.24-4.94) | <0.05 |  | 2.69(1.33-5.43) | <0.01 |

Model 1 adjusted for sex and age. Model 2 adjusted for sex, age, and arthritis.
