# Supplementary material for: Gender-specific associations between fat mass, metabolic syndrome and musculoskeletal pain in community residents: A three-year longitudinal study
Source: PLoS One. 2018 Jul 9;13(7):e0200138. doi: 10.1371/journal.pone.0200138 (PMC6037368; doi:10.1371/journal.pone.0200138)
Supplement: S4 Table — (DOCX) [file pone.0200138.s004.docx]

Supplementary Table 4. Distribution of new pain after 3 years in 4 groups classified by the presence and absence of metabolic syndrome and of obesity

|  | Group | No pain | New pain | p-value |
| --- | --- | --- | --- | --- |
| All | BMI < 25 & MS ≤ 2 | 173(66.0%) | 89(34.0%) | <0.05 |
|  | BMI < 25 & MS ≥ 3 | 54(57.4%) | 40(42.6%) |  |
|  | BMI ≥ 25 & MS ≤ 2 | 38(46.9%) | 43(53.1%) |  |
|  | BMI ≥ 25 & MS ≥ 3 | 57(56.4%) | 44(43.6%) |  |
|  | Total | 322(59.9%) | 216(40.1%) |  |
| Male | BMI < 25 & MS ≤ 2 | 105(68.2%) | 49(31.8%) | 0.462 |
|  | BMI < 25 & MS ≥ 3 | 25(64.1%) | 14(35.9%) |  |
|  | BMI ≥ 25 & MS ≤ 2 | 25(55.6%) | 20(44.4%) |  |
|  | BMI ≥ 25 & MS ≥ 3 | 41(67.2%) | 20(32.8%) |  |
|  | Total | 196(65.6%) | 103(34.4%) |  |
| Female | BMI < 25 & MS ≤ 2 | 68(63.0%) | 40(37.0%) | <0.05 |
|  | BMI < 25 & MS ≥ 3 | 29(52.7%) | 26(47.3%) |  |
|  | BMI ≥ 25 & MS ≤ 2 | 13(36.1%) | 23(63.9%) |  |
|  | BMI ≥ 25 & MS ≥ 3 | 16(40.0%) | 24(60.0%) |  |
|  | Total | 126(52.7%) | 113(47.3%) |  |

Definition of MONW: normal BMI range (18.5 to 25 kg/m2) and presence of ≥ 3 metabolic S features

Values are the number (%)**.P values (for trend) were determined by Pearson’s chi-square test.**
