# Supplementary material for: Gender-specific associations between fat mass, metabolic syndrome and musculoskeletal pain in community residents: A three-year longitudinal study
Source: PLoS One. 2018 Jul 9;13(7):e0200138. doi: 10.1371/journal.pone.0200138 (PMC6037368; doi:10.1371/journal.pone.0200138)
Supplement: S5 Table — (DOCX) [file pone.0200138.s005.docx]

Supplementary Table 5. Correlation between fat mass and pain after adjustment for age ( no pain/resolved pain group vs persistent pain group)

|  | resolved No pain group (N = 618) | persistent pain group  (N = 304) | p-value |
| --- | --- | --- | --- |
|  | Mean ± SE | Mean ± SE |  |
| All |  |  |  |
| Total fat mass, kg | 16.1384±0.2572 | 18.2095±0.3679 | <0.001 |
| Total lean mass, kg | 43.3814±0.3323 | 41.5459±0.4753 | 0.002 |
| Fat/muscle ratio | 0.3893±0.0073 | 0.4582±0.0105 | <0.001 |
| Male |  |  |  |
| Total fat mass, kg | 13.6971±0.3285 | 14.3869±0.5465 | 0.281 |
| Total lean mass, kg | 50.4179±0.3119 | 50.0200±0.5189 | 0.512 |
| Fat/muscle ratio | 0.2696±0.0061 | 0.2864±0.0102 | 0.159 |
| Female |  |  |  |
| Total fat mass, kg | 18.5656±0.3351 | 20.4622±0.4268 | 0.001 |
| Total lean mass, kg | 36.3963±0.2054 | 36.5200±0.2616 | 0.712 |
| Fat/muscle ratio | 0.5086±0.0086 | 0.5591±0.0109 | <0.001 |
