# Supplementary material for: Gender-specific associations between fat mass, metabolic syndrome and musculoskeletal pain in community residents: A three-year longitudinal study
Source: PLoS One. 2018 Jul 9;13(7):e0200138. doi: 10.1371/journal.pone.0200138 (PMC6037368; doi:10.1371/journal.pone.0200138)
Supplement: S6 Table — (DOCX) [file pone.0200138.s006.docx]

Supplementary Table 6. Association between each quartile of fat/muscle mass ratio and pain( no pain/resolved pain group vs persistent pain group)

|  | Crude | | Model 1 | | Model 2 | |
| --- | --- | --- | --- | --- | --- | --- |
| Fat/muscle mass ratio | OR (95% CI) | *P* | OR (95% CI) | *P* | OR (95% CI) | *P* |
| Quartile 1 | - | - | - | - | - | - |
| Quartile 2 | 1.10(0.73~1.64) | 0.661 | 1.08(0.71~1.64) | 0.722 | 1.09(0.71~1.65) | 0.702 |
| Quartile 3 | 1.75(1.17~2.60) | 0.006 | 1.58(0.96~2.59) | 0.074 | 1.56(0.94~2.56) | 0.083 |
| Quartile 4 | 2.18(1.46~3.25) | 0.000 | 1.94(1.13~3.33) | 0.017 | 1.83(1.06~3.17) | 0.030 |

Model 1 adjusted for sex and age. Model 2 adjusted for sex, age, and arthritis.
