# Supplementary material for: Gender-specific associations between fat mass, metabolic syndrome and musculoskeletal pain in community residents: A three-year longitudinal study
Source: PLoS One. 2018 Jul 9;13(7):e0200138. doi: 10.1371/journal.pone.0200138 (PMC6037368; doi:10.1371/journal.pone.0200138)
Supplement: S7 Table — (DOCX) [file pone.0200138.s007.docx]

Supplementary Table 7. Correlation between fat mass and pain after adjustment for age ( resolved pain group vs persistent pain group)

|  | resolved pain group  (N=296) | persistent pain group  (N=304) | p-value |
| --- | --- | --- | --- |
|  | Mean ± SE | Mean ± SE |  |
| All |  |  |  |
| Total fat mass, kg | 17.1720±0.3892 | 18.1402±0.3840 | 0.078 |
| Total lean mass, kg | 41.9056±0.4644 | 41.4501±0.4582 | 0.487 |
| Fat/muscle ratio | 0.4285±0.0112 | 0.4573±0.0110 | 0.068 |
| Male |  |  |  |
| Total fat mass, kg | 13.4581±0.5421 | 14.3461±0.5445 | 0.250 |
| Total lean mass, kg | 50.5437±0.5286 | 49.9805±0.5309 | 0.454 |
| Fat/muscle ratio | 0.2637±0.0099 | 0.2858±0.0100 | 0.119 |
| Female |  |  |  |
| Total fat mass, kg | 19.4308±0.4545 | 20.3863±0.4437 | 0.135 |
| Total lean mass, kg | 36.6504±0.2683 | 36.3989±0.2619 | 0.504 |
| Fat/muscle ratio | 0.5287±0.0117 | 0.5587±0.0114 | 0.068 |
