# Supplementary material for: Gender-specific associations between fat mass, metabolic syndrome and musculoskeletal pain in community residents: A three-year longitudinal study
Source: PLoS One. 2018 Jul 9;13(7):e0200138. doi: 10.1371/journal.pone.0200138 (PMC6037368; doi:10.1371/journal.pone.0200138)
Supplement: S8 Table — (DOCX) [file pone.0200138.s008.docx]

Supplementary Table 8. Association between each quartile of fat/muscle mass ratio and pain ( resolved pain group vs persistent pain group)

|  | Crude | | Model 1 | | Model 2 | |
| --- | --- | --- | --- | --- | --- | --- |
| Fat/muscle mass ratio | OR (95% CI) | *P* | OR (95% CI) | *P* | OR (95% CI) | *P* |
| Quartile 1 | - | - | - | - | - | - |
| Quartile 2 | 0.99(0.61~1.60) | 0.960 | 1.06(0.64~1.74) | 0.833 | 1.06(0.64~1.74) | 0.833 |
| Quartile 3 | 1.38(0.86~2.21) | 0.184 | 1.65(0.91~2.99) | 0.098 | 1.66(0.92~3.01) | 0.095 |
| Quartile 4 | 1.23(0.77~1.94) | 0.386 | 1.55(0.82~2.91) | 0.176 | 1.57(0.83~2.98) | 0.164 |

Model 1 adjusted for sex and age. Model 2 adjusted for sex, age, and arthritis.
